# The Effect of Hydrophilic Ionic Liquids 1-Ethyl-3-Methylimidazolium Lactate and Choline Lactate on Lipid Vesicle Fusion

**DOI:** 10.1371/journal.pone.0085467

**Published:** 2013-12-31

**Authors:** Eri H. Hayakawa, Eiko Mochizuki, Tetsuya Tsuda, Kazunari Akiyoshi, Hiroyuki Matsuoka, Susumu Kuwabata

**Affiliations:** 1 Laboratory of Medical Zoology and Parasitology, Department of Infection and Immunity, Jichi Medical University, Shimotsuke, Tochigi, Japan; 2 Department of Applied Chemistry, Graduate School of Engineering, Osaka University, Suita, Osaka, Japan; 3 Department of Polymer Chemistry, Graduate School of Engineering, Kyoto University, Kyoto, Kyoto, Japan; 4 Japan Science and Technology Agency, ERATO, Kawaguchi, Saitama, Japan; 5 Japan Science and Technology Agency, CREST, Kawaguchi, Saitama, Japan; Aligarh Muslim University, India

## Abstract

Ionic liquids (ILs) are room-temperature molten salts that have applications in both physical sciences and more recently in the purification of proteins and lipids, gene transfection and sample preparation for electron microscopy (EM) studies. Transfection of genes into cells requires membrane fusion between the cell membrane and the transfection reagent, thus, ILs may be induce a membrane fusion event. To clarify the behavior of ILs with cell membranes the effect of ILs on model membranes, i.e., liposomes, were investigated. We used two standard ILs, 1-ethyl-3-methylimidazolium lactate ([EMI][Lac]) and choline lactate ([Ch][Lac]), and focused on whether these ILs can induce lipid vesicle fusion. Fluorescence resonance energy transfer and dynamic light scattering were employed to determine whether the ILs induced vesicle fusion. Vesicle solutions at low IL concentrations showed negligible fusion when compared with the controls in the absence of ILs. At concentrations of 30% (v/v), both types of ILs induced vesicle fusion up to 1.3 and 1.6 times the fluorescence intensity of the control in the presence of [Ch][Lac] and [EMI][Lac], respectively. This is the first demonstration that [EMI][Lac] and [Ch][Lac] induce vesicle fusion at high IL concentrations and this observation should have a significant influence on basic biophysical studies. Conversely, the ability to avoid vesicle fusion at low IL concentrations is clearly advantageous for EM studies of lipid samples and cells. This new information describing IL-lipid membrane interactions should impact EM observations examining cell morphology.

## Introduction

 Ionic liquids (ILs), molten salts with low melting temperatures (<100 °C), are a complex of anionic and cationic ions. ILs have unique characteristics, including high ionic conductivity, a wide range of viscosities and are nonvolatile. These features make ILs suitable for many applications such as surface finishing, electrochemical deposition, electroplating and formation of carbon nanotubes. Recently, ILs have been used in analytical biochemistry examining proteins and lipids by replacing conventionally used organic solvents such as methanol/ chloroform and acetonitrile. For example, ILs provide matrices for matrix-assisted laser desorption and ionization (MALDI) of biomolecules in mass spectroscopy [1] and they improve the homogeneity of ionized molecules and ion yield [2,3]. ILs have also been used to enhance the separation of proteins [4] in sodium dodecyl sulfate polyacrylamide gel electrophoresis (SDS-PAGE), chromatography techniques including high performance liquid chromatography (HPLC) [5], hydrophobic interaction chromatograph [6], gas chromatography [7,8]. Furthermore, if suitable preparation conditions can be found that ensure regular, high quality crystals then various soluble and transmembrane proteins can be examined, thus promoting protein structure-function studies. However, preparing high quality protein crystals with good diffraction properties for better 3D structure estimation is often a difficult task. In this point, ILs also can provide better conditions for protein crystallization [9]. In addition, ILs have been studied as gene transfection reagents [10]. This application suggests that ILs may also aid membrane fusion.

 Polyethylene glycols (PEGs) show features that are similar to the interactions that ILs make with biomolecules. PEGs are polymers of ethylene glycol and have average molecular weights of 180−3,500,000. PEG has been a common reagent used for protein precipitation and purification of proteins by liquid chromatography-mass spectrometry, because of their ability to induce liquid biphases [4]. PEG improves the protein crystallization process by eliminating water between protein molecules and the inner structures of proteins. PEGs are also useful for purification of antibodies [11], DNA precipitation [12] and are also used as carriers for DNA transfection of cells [13], initiators of membrane fusion between cells [14,15] and lipid vesicle-vesicle fusion [16]. 

 Since PEGs and ILs share similarities in influencing intermolecular interactions, ILs may induce membrane fusion, the possibility can be predicted based on the observed properties to improve ILs-lipid molecules interactions. In this study, we have used fluorescence resonance energy transfer (FRET) and dynamic light scattering (DLS) to study the interaction of ILs with model liposome membranes. The ILs we used were part of the lactate ([Lac])-series ILs, namely the commonly used 1-ethyl-3-methylimidazolium lactate ([EMI][Lac]) and choline lactate ([Ch][Lac]). Our data demonstrated that ILs can induce lipid vesicle fusion under high IL concentrations without lipid vesicle aggregation.

## Materials and Methods

### Materials

1-Ethyl-3-methylimidazolium lactate ([EMI] [CH_3_(OH)CHCOO]) and choline lactate ([Ch] [CH_3_(OH)CHCOO]) were synthesized and purified as reported previously [17]. 1,2-Dioleoyl-*sn*-glycero-3-phosphocholine (DOPC), cholesterol (chol), 1,2-dipalmitoyl-*sn*-glycero-3-phosphoethanolamine-N-(Lissamine Rhodamine B Sulfonyl) (N-Rh-PE) were purchased from Avanti Polar Lipids (Alabaster, AL). Sphingomyelin (SM; chicken egg) were purchased from Avanti Polar Lipids and Sigma (Saint Louis, MO). *N-*(7-Nitrobenz-2-Oxa-1,3-Diazol-4-yl)-1,2-dihexadecanoyl-sn-glycero-3-phosphoethanolamine, triethylammonium salt (N-NBD-PE) was purchased from Invitrogen (Eugene, OR). Reagent grade chloroform and methanol were purchased from Wako (Osaka, Japan). 

### Lipid vesicle preparation

Large unilamellar vesicles were prepared according to established methods [18]. To 1:1 (mol/mol %) DOPC/SM in a 3:1 chloroform/methanol solution was added 30 mol% chol of DOPC+SM and 0.5 mol % of N-NBD-PE and N-Rh-PE. After the solvent was removed by a stream of N_2_ gas, the lipids were dried overnight in a vacuum and then hydrated in a phosphate buffer (pH 7.4) at 50−55 °C for 1 h and converted to multilamellar vesicles by rigorous mixing. Large unilamellar vesicles were prepared from this solution by 21 extrusion cycles at 55 °C with an Avanti Polar Lipid extruder equipped with a 100-nm pore size polycarbonate membrane (Whatman, Florham Park, NJ). The final lipid concentration was 0.52 mM.

### Fluorescence resonance energy transfer (FRET) analysis

FRET signals were detected by a Hitachi F4500 fluorescence spectrophotometer (Hitachi High-Technologies Corporation, Tokyo, Japan), and measurements were carried out as described previously [18–20]. Each sample consisted of two types of DOPC-SM-chol unilamellar vesicles. One of the vesicles contained two fluorescent probes (0.5 mol% of N-NBD-PE and N-Rh-PE), whereas the other vesicles contained no FRET probes. The FRET donor NBD-PE was excited at 473 nm and the emission intensity of the Rho-PE as the acceptor were monitored at 580 nm. All FRET measurements were carried out at room temperature (25–26 °C), as controlled by a recirculating chiller (Cool Ace CAE-1000A, Tokyo Rikakikai Co., Ltd., Tokyo, Japan). 

### Dynamic light scattering (DLS) measurements

Lipid vesicle sizes were obtained by 633 nm light scattering with a Malvern Zetasizer Nano series zen3600 (Malvern Instruments Ltd., Worcestershire, UK) at room temperature. We measured unilamellar vesicle sizes in the absence of ILs, followed by measurements at various concentrations of ILs. We averaged nine independent measurements of vesicle sizes, where each measurement was a mean of 13−15 readings by the instrument. All data were analyzed by the Zetasizer software (Malvern Instruments Ltd.). To correct the DLS data for changes in viscosity, we used a DV-II+ Pro viscometer (Brookfield Engineering Laboratories, Boston, MA) and measured the viscosity of the ILs at each concentration (10, 20 and 30%) and at room temperature. The torque conditions were set at 60 and 100 rpm, and we confirmed there was no difference between 60 and 100 rpm due to the IL viscosities. Size distributions of liposomes were fitted with the Origin ver.7.5J software (Origin Lab, Northampton, MA). 

## Results

### ILs-induced vesicle fusion detected by FRET

**Figure 1:**
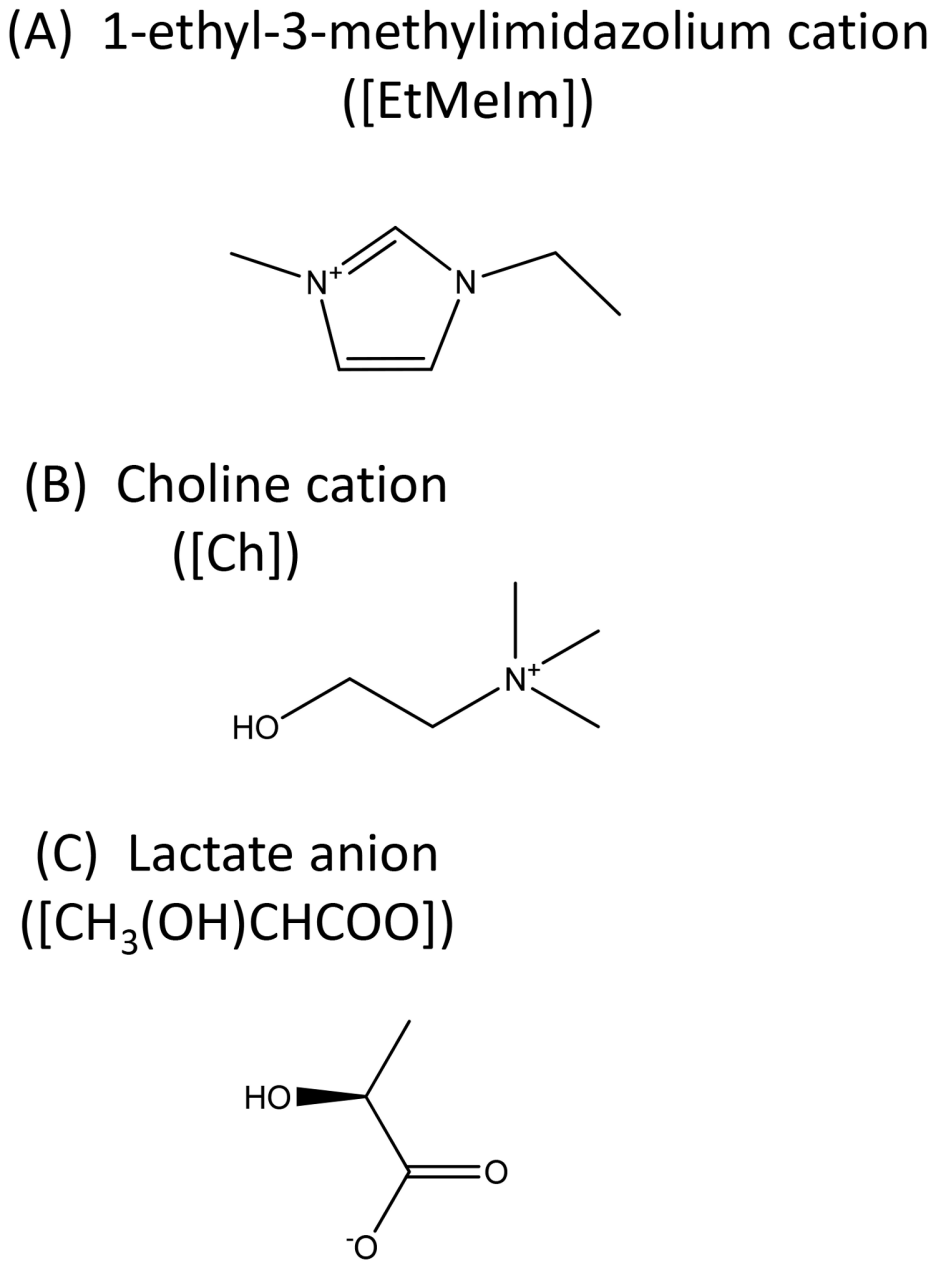
Structure of ionic liquids (ILs). Schematic of the ion groups [EMI], [Ch] and [Lac]. [EMI][Lac] and [Ch][Lac] have different cationic groups.

**Figure 2:**
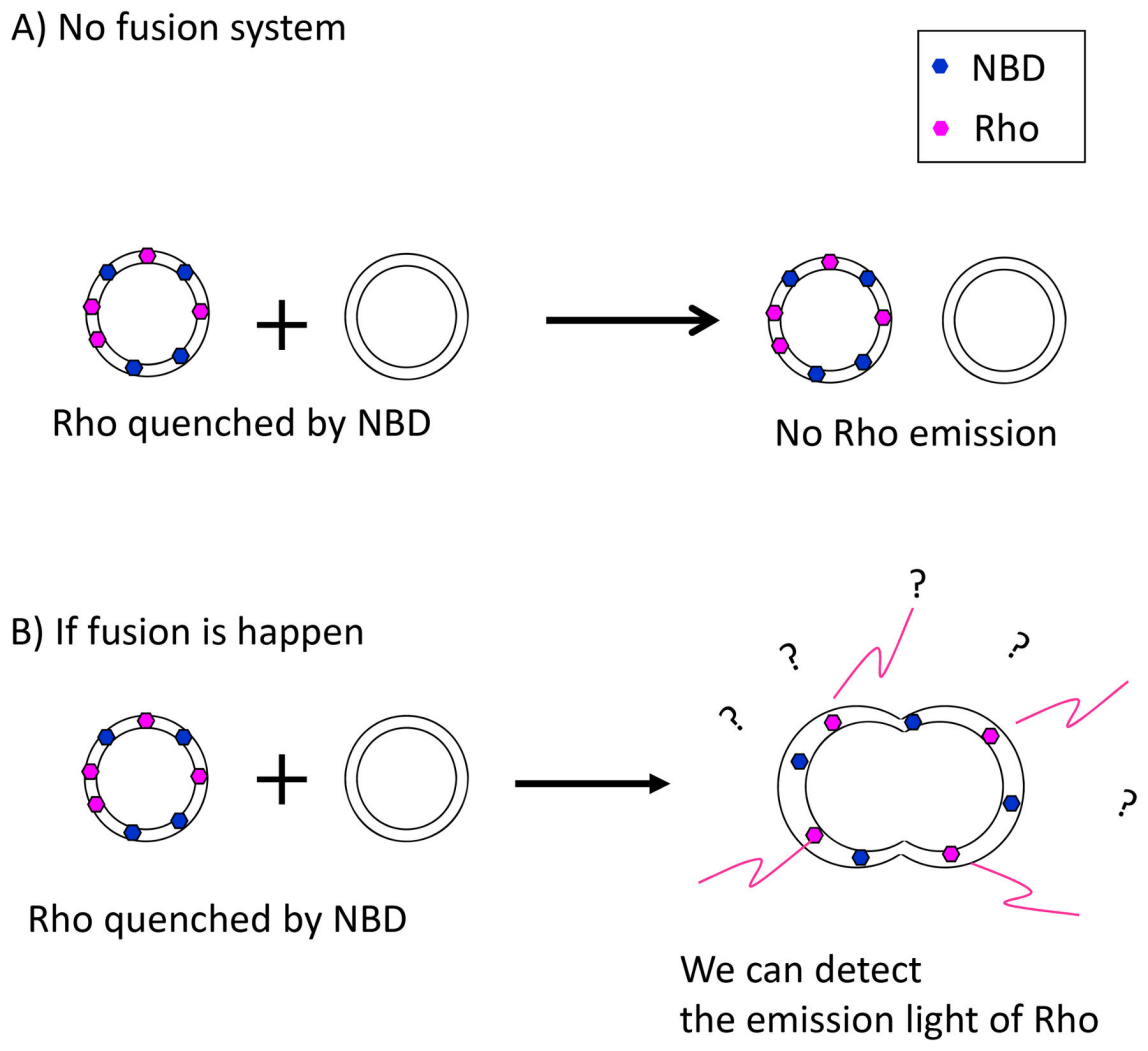
Schematic of the FRET mechanism with respect to vesicle fusion. (A) When the fluorescent probes NBD-PE and Rho-PE are in a single vesicle, the distance between them is very short, causing fluorescence quenching and no energy transfer. (B) When a probe-labeled vesicle fuses with a non-labeled vesicle, the membrane surface area increases significantly. Thus, the larger distance between NBD-PE and Rho-PE is optimal for fluorescence energy transfer and emission from Rho-PE is observed. Note that the fusion of non-labeled vesicles is not undetected. Furthermore, if labeled lipid vesicles fuse, emission depends on the distances between the NBD-PE and Rho-PE probes.

Figure 1 depicts the anionic and cationic structures of the [EMI][Lac] and [Ch][Lac] ILs used in this study and Figure 2 describes the FRET mechanism which is presented in the Materials and Methods section.

**Figure 3:**
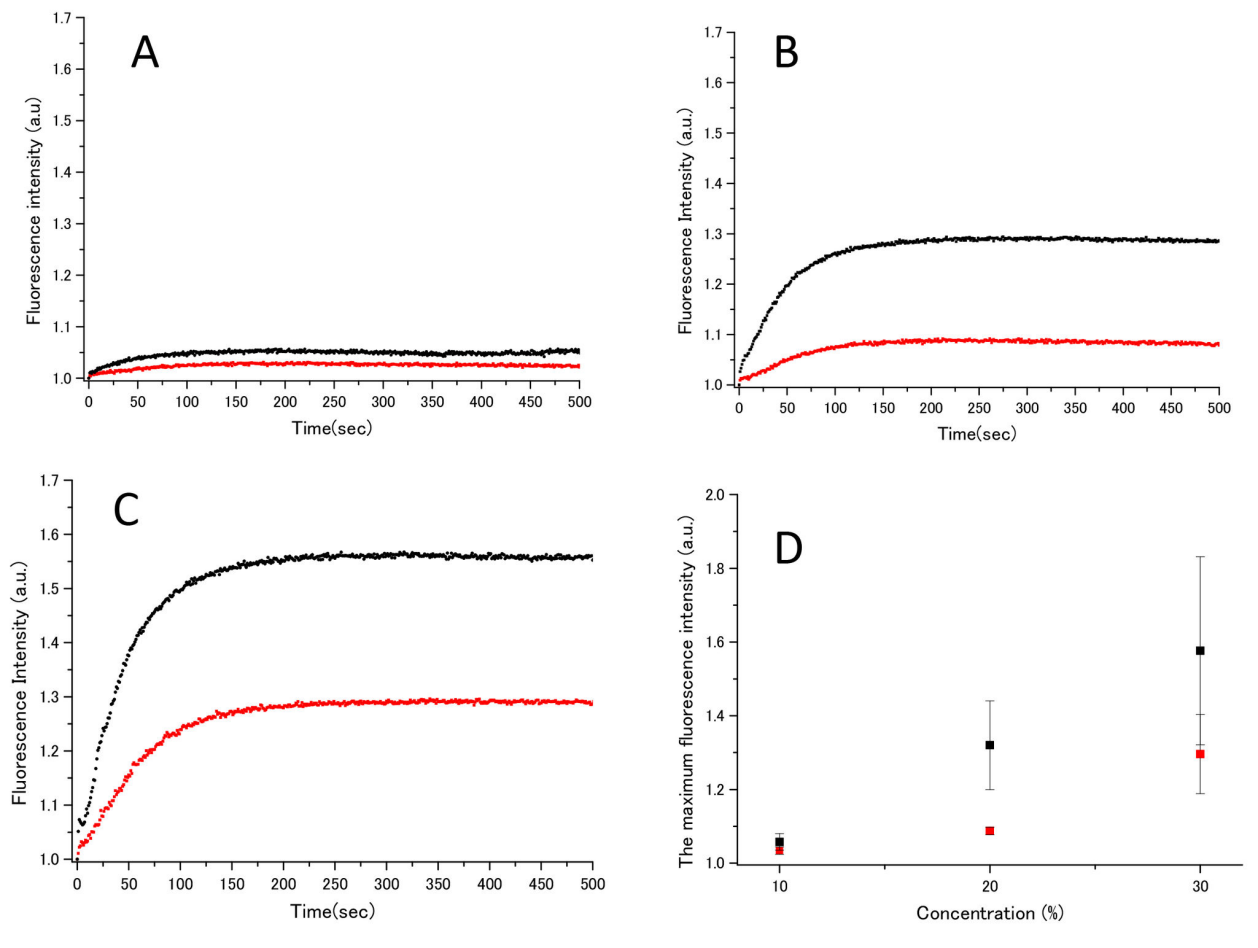
Effect of ILs on the FRET ratio for DOPC-SM-Chol bilayer vesicles. [EMI][Lac] and [Ch][Lac] were used at 10% (A), 20% (B) and 30% (v/v) (C). For the 20% and 30% concentrations, [EMI][Lac] (black line) shows a higher ability to induce membrane fusion than [Ch][Lac] (red line). However, there were no distinct differences between [EMI][Lac] and [Ch][Lac] at a concentration of 10% (v/v) (A). (D) Shows the maximum FRET fluorescence intensity changes (normalized) as a function of [EMI][Lac] and [Ch][Lac] concentrations. At 30%-[EMI][Lac] (black square), the ratio of membrane fusion ability was ≈1.4 times higher than that of [Ch][Lac] (red square) at the same concentration.

Normally, we cannot detect Rho-PE light emission because of quenching caused by the close proximity of the NBD-PEs and Rho-PEs within the same vesicle membrane (Figure 2A). However, if vesicle fusion occurs between the labeled and unlabeled vesicles, the distance between the NBD-PE and Rho-PE is increased by the expansion of the vesicle surface area and the NBD-PE energized Rho-PE emission is no longer quenched. Thus, we observe an increase in Rho-PE emission due to IL-induced vesicle fusion (Figure 2B). We investigated lipid membrane (vesicle) fusion by FRET at IL concentrations (volume/volume) of 10% (Figure 3A), 20% (Figure 3B) and 30% (Figure 3C). At 10% (v/v) of [EMI][Lac] (black line), no significant difference in the FRET signal was observed. However, for the 20% and 30% IL concentrations (Figure 3B, C), we observed that [EMI][Lac] showed higher fluorescence intensities than [Ch][Lac]. Figure 3D plots the maximum fluorescence intensity values (plateaus) at each concentration of the ILs. These data demonstrate that both [EMI][Lac] and [Ch][Lac] induce vesicle fusion, and that [EMI][Lac] is more effective than [Ch][Lac]. The FRET intensity remained constant when the maximum levels were reached, with only small fluctuations (data not shown). The time delay for the increase in fluorescence intensity following the addition of ILs to the vesicle solutions varied owing to the vesicle fusion process. Thus, the fluorescence intensity without ILs was measured first as a control and then the intensities in the presence of ILs were measured. The fluorescence intensities were averaged and normalized with the control intensities.

### The effect of ILs on vesicle sizes by DLS measurements

Increased FRET was observed because of the expanded surface area of the fused vesicles and the distance of NBD-PE and Rho-PE are applicable for fluorescence energy transfer. To eliminate the possibility that FRET occurred because of a possible phase transition induced by ILs added to the vesicle solution, we used DLS to determine the average vesicle size before and after the addition of the ILs. The viscosities of [EMI][Lac] were 1.49, 2.08 and 3.02, and those of [Ch][Lac] were 1.40, 1.73 and 2.28 at 10, 20 and 30% (v/v), respectively.

**Figure 4:**
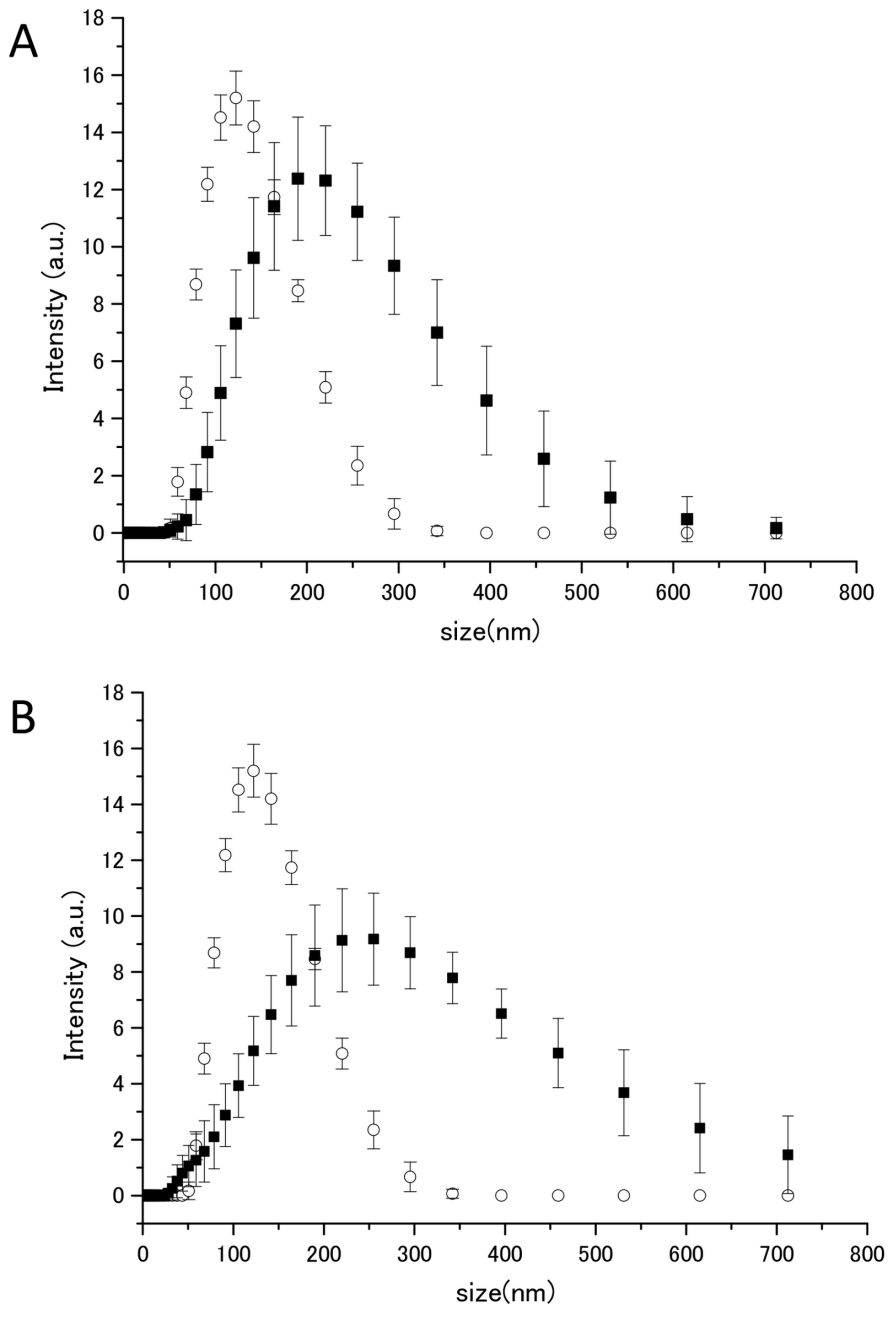
Comparison of vesicle diameters with and without 30% [Ch][Lac] and [EMI][Lac]. 30%-[Ch][Lac] (A) and 30%-[EMI][Lac] (B), as detected by DLS. The circles indicate the vesicle diameter distribution before the addition of the ILs. For [Ch][Lac], the peak diameter increased from 122.4 to 220.5 nm, and for [EMI][Lac], the peak increased to 255 nm. Furthermore, the population of vesicle diameters larger than 400 nm was greater for [EMI][Lac] than for [Ch][Lac].

**Figure 5:**
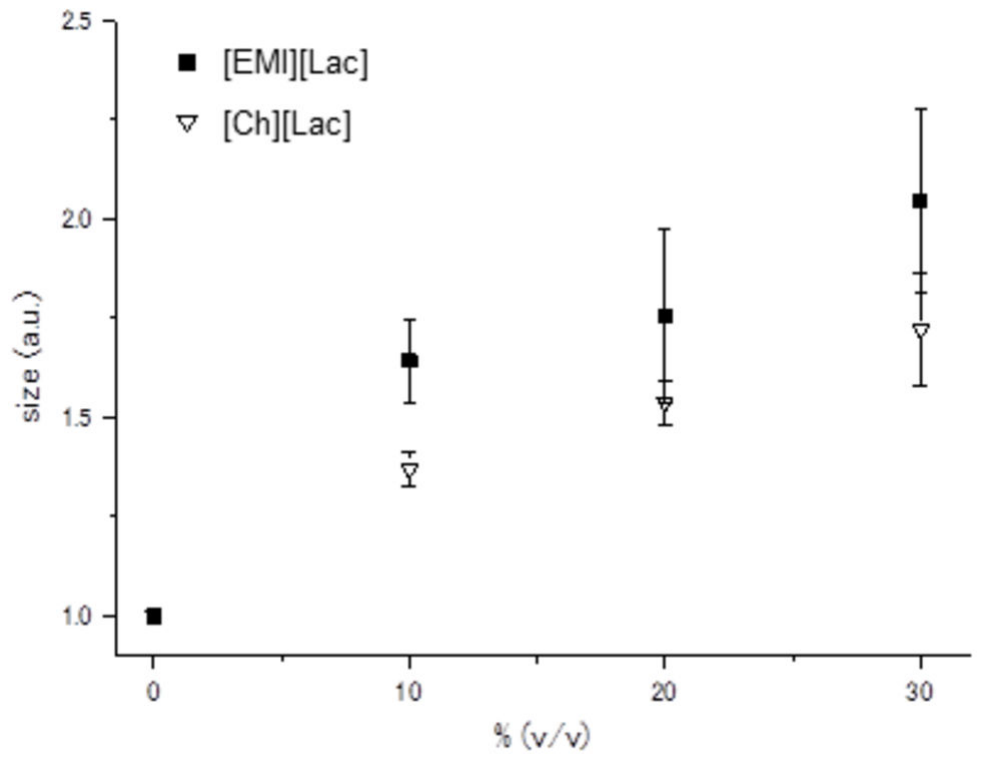
Comparison of the vesicle diameters at various concentration of [Ch][Lac] and [EMI][Lac]. Comparison of the vesicle sizes between control vesicles and the presence of 30%-[EMI][Lac] or [Ch][Lac] (see Supporting Data). For both ILs, peak sizes became larger with increasing IL concentration.

 In Figure 4, the circles indicate the vesicle diameter distribution before the addition of the ILs; the peak of the distribution is 122.4 nm (Figures 4A, B). In the presence of 30%- [Ch][Lac] and [EMI][Lac], the peaks of the distributions increase to 220.5 nm (Figure 4A, black square) and 255 nm (Figure 4B, black square)), respectively. When the concentrations of both ILs were increased from 10 to 30%, the vesicle diameters increased (supporting data). This is depicted in Figure 5, which shows the relative increase in peak size of the vesicles as a function of IL concentrations. Viscosities of ILs used in this experiment are very low, thus changes in vesicle sizes were primarily due to the addition of ILs. The DLS data support the expansion of the vesicles sizes by ILs, as observed in the FRET experiments.

## Discussion

 We have demonstrated that at high concentrations two different ILs induced lipid membrane fusion. IL-water and PEG-water hydrogen bonding are key factors in understanding the vesicle fusion mechanisms. PEGs, [EMI][Lac] and [Ch][Lac] not only exhibit similar effects on protein crystallization, but they also have similar Kamlet-Taft parameters (α, β and π^*^) [21] (see Table 1). The α, β and π^*^ define the hydrogen bond acidity, hydrogen bond basicity and dipolarity/polarizability effects, respectively, in solvents. The Kamlet-Taft parameters for the ILs are given in Table 1 and are estimated by reference to literature. Detailed information describing these parameters are found in some literatures [22–24]. PEGs induce lipid membrane fusion via water interactions and when the β value of ILs is >0.7 the ILs show properties that are similar to each other. Thus, [EMI][Lac] and [Ch][Lac] probably interact with water via hydrogen bonding in a manner similar to PEGs. Generally, the molecular structure of an anion group has an effect on hydrogen bond donor ability [25]. In this study, we have used ILs with the same anion groups, so this feature is probably the reason that both ILs induced membrane fusion. However, [EMI][Lac] was found to show much greater ability to induce vesicle fusion than [Ch][Lac]. This observation may be due to the different cationic groups, which have different hydrogen bonding acceptor abilities (β values). 

**Table 1:** Comparison of the physico-chemical properties between PEG and ILs.

	α	β	π^*^	$E_{T}^{N}$
PEG-600	0.32	0.66	0.84	0.522
[Ch][Lac]	0.59	0.80	1.12	0.68
[EMI][Lac]	0.50	0.95	1.08	0.62

Comparison of Kamlet-Taft parameters for PEG, [EMI][Lac] and [Ch][Lac]. Values of α and β were very similar in all three materials, which implies that PEG, [EMI][Lac] and [Ch][Lac] exhibit similar hydrogen bonding forces. The π^*^ values are also similar, which indicates that the charge distributions are similar. From those properties, PEG, [EMI][Lac] and [Ch][Lac] should have similar water affinities.

 Since lipid vesicles exist in water, the interaction of vesicles with PEGs and ILs probably involves similar bonding interactions and may be the basis in which ILs induce vesicle fusion. PEGs absorb and remove water from the surface of vesicles. Consequently, the polar head groups of the outer lipids bond to each other. The inner lipids of the bilayer then fuse, followed by total membrane fusion [26]. If this mechanism is applicable to ILs, they must be in close proximity of the lipid polar head group-water interface. Evans et al. reported that the ILs induce the formation of a cholesterol-water interface in 1-butyl-3-methylimidazolium chloride ([BMI]Cl) and 1-butyl-3-methylimidazolium bis (trifluoromethylsulfonyl)imide ([BMI][Tf_2_N]) in a cholesterol bilayer solution [27]. This occurs even though the ILs have a different affinity owing to the anionic structures. Despite the fact that [BMI]Cl has a high β value and [BMI] [Tf_2_N] has a low β value [28], both ILs form a cholesterol-water interface. These observations suggest that [EMI][Lac] and [Ch][Lac] will also localize around the lipid-water interface in a vesicle solution. Furthermore, another study reported that [EMI] ethyl sulfate in excess water interacts with water via the hydrogen atoms on the [EMI] imidazolium ring [29]. Taken together, [EMI][Lac] and [Ch][Lac] probably localize at the lipid bilayer-water interface, and by hydrogen-bonding, remove water from the surface of the vesicle. This then causes the polar portions of the lipid vesicles to bond, as discussed above. 

 The FRET data indicated that [EMI][Lac] has a greater ability to induce membrane fusion than [Ch][Lac] under high IL concentrations. In contrast, Figure 3 and Figure 4 revealed negligible vesicle fusion at low (10% v/v) concentrations for both ILs. Thus, the hydrogen bonding interaction between the C-H groups of [EMI] and water are weaker if there is excess water [29], and low concentrations of ILs will not absorb water surrounding the vesicles in sufficient quantities. Consequently, the probability of adhesion and fusion of the vesicles will be markedly reduced. Therefore, the ILs-water, ILs-lipid and lipid-water interactions depend on how much water is present. The subtle fluctuations in fluorescence intensities (data not shown) observed in the plateau region following the emission maxima may be due to perturbations of the lipid membrane caused by subtle changes in the hydrogen bonding forces in the ILs-water-vesicle interaction. 

 Since PEGs have been used as a regent to induce cells/membranes fusion, we measured how vesicle sizes changed by PEG (1.1mg/ml, [16]), [EMI][Lac], or [Ch][Lac] using DLS. However, in the presence of 3% (v/v) PEG in a liposome solution, the polydispersity index (PDI) showed over 0.4−1.0. This shows that PEGs transformed homogeneous lipid vesicle solutions to heterolytic and polydisperse systems, and this sample system is not suitable for DLS measurements. On the other hand, when [EMI][Lac] or [Ch][Lac] were mixed with lipid vesicle solutions, the PDI values were under 0.4 (primarily between 0.15−0.33) and we could carry out the DLS measurements successfully. This comparison also indicated PEGs cause significant aggregation with vesicles in their cluster. In contrast, both ILs used in this study do not induce aggregation like PEGs, even both ILs enlarged the size of the liposomes. These phenomena of PEG support the previous observation that PEGs cause aggregation of vesicles as an early process of phospholipid vesicle fusion [30] . 

**Figure 6:**
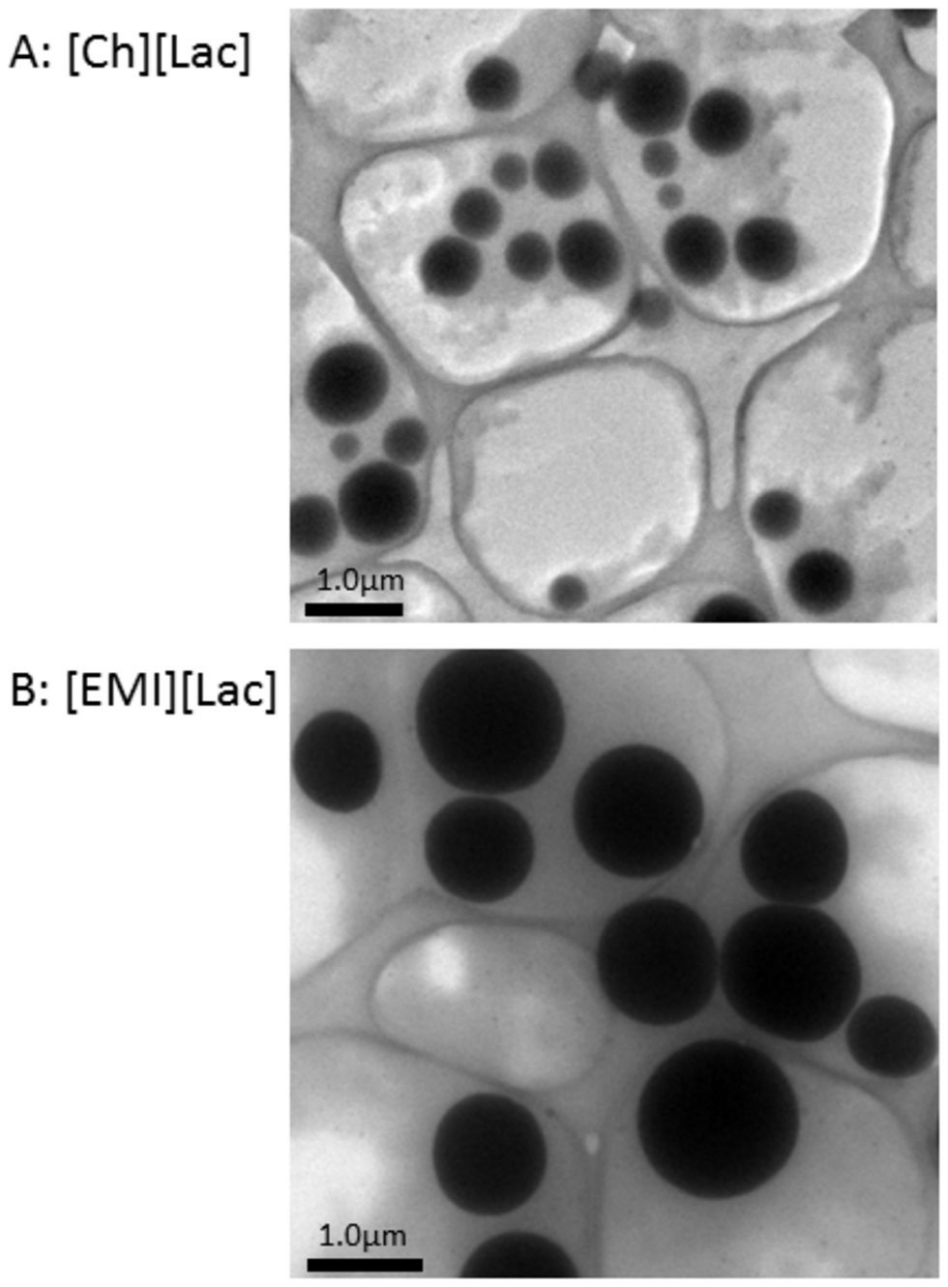
TEM imaging of liposomes with ILs and PEG. After the addition of each [Ch][Lac] and [EMI][Lac] to liposome solutions, and lipids composition and ratio of liposomes were same with FRET and DLS measurements. Samples were put on mesh for EM, and the removal of excess water/sample was carried out. Samples were then set into the TEM. In the case of [Ch][Lac]-liposomes (A), there are various sizes of liposomes between 340−780 nm. In the case of [EMI][Lac]-liposomes (B), liposomes became larger than [Ch][Lac]-liposomes, and the sizes were approximately 1−1.7 μm. Both of the IL-liposomes shapes remained circular.

 Recently, ILs have been used for preparing samples for electron microscopy (EM). ILs can prevent the electrization of samples by an electrification phenomenon known as “charge-up” on the surface of biological samples. This problem can be remedied by a simple procedure that introduces ILs to biomaterials to the sample, which allows significantly shorter preparation times. In addition, time-consuming metal coating procedures with Pt and/or C are no longer required when ILs are used. These benefits expand applications and increase the efficiency of sample preparations. Figure 6 shows TEM images using [EMI][Lac]- and [Ch][Lac]-liposome. Liposomes were extruded through a 100 nm pore membrane. Since the TEM environment is under a vacuum, it was not possible to directly compare the FRET, DLS and TEM imaging data under equivalent conditions. We found both ILs enlarged liposome sizes in TEM imaging, and the vesicle morphology was kept under vacuum conditions. From these additional results, the ILs that we used in this study should be available for liposome imaging by TEM. 

## Conclusions

 This study is the first to report that high concentrations of ILs induce lipid membrane fusion without aggregation, as detected by both FRET and DLS. We found both ILs also enlarged liposome size by TEM. However, low concentrations of ILs hardly affected the vesicles and should represent suitable conditions for artifact-free preparation of EM samples [17]. EM is becoming a powerful tool for cell biology by imaging surface and intracellular structures at high resolution. Since ILs have high electrical conductivity [31], they behave like metal coatings for the imaging of cells. Furthermore, ILs emit secondary electrons more readily than metals, thus enabling secondary electron images without sample charging. Biological sample preparations are challenging, so it is anticipated that ILs will improve these technical issues associated with EM. Wider applications of ILs with a better understanding of their interactions with biomolecules could open new areas in the field of biological sciences. Therefore, future studies will investigate the relationship between ILs and biomaterials or biomolecules in order to expand the potential of EM. 

## Supporting Information

Figure S1 **Size distributions of vesicles under various concentrations of ILs.** The IL concentrations were 10, 20 and 30% (v/v) in the vesicle solution. For both ILs, the maximum size of the vesicles increased with increasing IL concentration. In addition, the width of the size distribution became broader under increasing IL concentrations.
(TIF)

## References

[1] Ahmad F, Wu HF (2011) Characterization of pathogenic bacteria using ionic liquid via single drop microextraction combined with MALDI-TOF MS. Analyst 136: 4020-4027. doi:10.1039/c1an15350a. PubMed: 21804984. 21804984

[2] Fitzgerald JJ, Kunnath P, Walker AV (2010) Matrix-enhanced secondary ion mass spectrometry (ME SIMS) using room temperature ionic liquid matrices. Anal Chem 82: 4413-4419. doi:10.1021/ac100133c. PubMed: 20462181. 20462181

[3] Li YL, Gross ML, Hsu FF (2005) Ionic-liquid matrices for improved analysis of phospholipids by MALDI-TOF mass spectrometry. J Am Soc Mass Spectrom 16: 679-682. PubMed: 15862769. 15862769 10.1016/j.jasms.2005.01.017

[4] Li J, Rajagopalan R, Jiang J (2008) Polymer-induced phase separation and crystallization in immunoglobulin G solutions. J Chem Phys 128: 205105. doi:10.1063/1.2919565. PubMed: 18513048. 18513048

[5] Suh JH, Lee YY, Lee HJ, Kang M, Hur Y et al. (2013) Dispersive liquid-liquid microextraction based on solidification of floating organic droplets followed by high performance liquid chromatography for the determination of duloxetine in human plasma. J Pharm Biomed Anal 75: 214-219. PubMed: 23277153. 23277153 10.1016/j.jpba.2012.11.041

[6] Murphy PJ, Stone OJ, Anderson ME (2011) Automated hydrophobic interaction chromatography column selection for use in protein purification. J Vis Exp: ([MedlinePgn:]) PubMed: 21968976. 10.3791/3060 PMC3230179 21968976

[7] Delmonte P, Fardin-Kia AR, Kramer JK, Mossoba MM, Sidisky L et al. (2012) Evaluation of highly polar ionic liquid gas chromatographic column for the determination of the fatty acids in milk fat. J Chromatogr A 1233: 137-146. doi:10.1016/j.chroma.2012.02.012. PubMed: 22386057. 22386057

[8] Cherta L, Portoles T, Beltran J, Pitarch E, Mol JG, et al. (2013) Application of gas chromatography-(triple quadrupole) mass spectrometry with atmospheric pressure chemical ionization for the determination of multiclass pesticides in fruits and vegetables. J Chromatogr A 1314C: 224-240 10.1016/j.chroma.2013.09.029 24070626

[9] Weingärtner H, Cabrele C, Herrmann C (2012) How ionic liquids can help to stabilize native proteins. Phys Chem Chem Phys 14: 415-426. doi:10.1039/c1cp21947b. PubMed: 22089969. 22089969

[10] Zhang Y, Chen X, Lan J, You J, Chen L (2009) Synthesis and biological applications of imidazolium-based polymerized ionic liquid as a gene delivery vector. Chem Biol Drug Des 74: 282-288. doi:10.1111/j.1747-0285.2009.00858.x. PubMed: 19703030. 19703030

[11] Giese G, Myrold A, Gorrell J, Persson J (2013) Purification of antibodies by precipitating impurities using Polyethylene Glycol to enable a two chromatography step process. J Chromatogr B Analyt Technol Biomed Life Sci 938C: 14-21. PubMed: 24036248. 10.1016/j.jchromb.2013.08.029 24036248

[12] He Z, Zhu Y, Gu H (2013) A new method for the determination of critical polyethylene glycol concentration for selective precipitation of DNA fragments. Appl Microbiol Biotechnol 97: 9175-9183. PubMed: 23982329. 23982329 10.1007/s00253-013-5195-0

[13] Schmieder AH, Grabski LE, Moore NM, Dempsey LA, Sakiyama-Elbert SE (2007) Development of novel poly(ethylene glycol)-based vehicles for gene delivery. Biotechnol Bioeng 96: 967-976. PubMed: 17039465. 17039465 10.1002/bit.21199

[14] Li LH, Hui SW (1994) Characterization of PEG-mediated electrofusion of human erythrocytes. Biophys J 67: 2361-2366. doi:10.1016/S0006-3495(94)80722-2. PubMed: 7696475. 7696475 PMC1225620

[15] Pedrazzoli F, Chrysantzas I, Dezzani L, Rosti V, Vincitorio M et al. (2011) Cell fusion in tumor progression: the isolation of cell fusion products by physical methods. Cancer Cell Int 11: 32 PubMed: 21933375. 21933375 10.1186/1475-2867-11-32 PMC3187729

[16] Weinreb G, Lentz BR (2007) Analysis of membrane fusion as a two-state sequential process: evaluation of the stalk model. Biophys J 92: 4012-4029. doi:10.1529/biophysj.106.090043. PubMed: 17369418. 17369418 PMC1869000

[17] Tsuda T, Nemoto N, Kawakami K, Mochizuki E, Kishida S et al. (2011) SEM observation of wet biological specimens pretreated with room-temperature ionic liquid. Chembiochem 12: 2547-2550. PubMed: 21990115. 21990115 10.1002/cbic.201100476

[18] Hayakawa E, Tokumasu F, Nardone GA, Jin AJ, Hackley VA et al. (2007) A Mycobacterium tuberculosis-derived lipid inhibits membrane fusion by modulating lipid membrane domains. Biophys J 93: 4018-4030. PubMed: 17704144. 17704144 10.1529/biophysj.107.104075 PMC2084222

[19] Lentz BR (1994) Polymer-induced membrane fusion: potential mechanism and relation to cell fusion events. Chem Phys Lipids 73: 91-106. doi:10.1016/0009-3084(94)90176-7. PubMed: 8001186. 8001186

[20] Malinin VS, Haque ME, Lentz BR (2001) The rate of lipid transfer during fusion depends on the structure of fluorescent lipid probes: a new chain-labeled lipid transfer probe pair. Biochemistry 40: 8292-8299. doi:10.1021/bi010570r. PubMed: 11444975. 11444975

[21] Jessop PG, Jessop DA, Fu DB, Phan L (2012) Solvatochromic parameters for solvents of interest in green chemistry. Green Chemistry 14: 1245-1259. doi:10.1039/c2gc16670d.

[22] Lee JM, Ruckes S, Prausnitz JM (2008) Solvent polarities and kamlet-taft parameters for ionic liquids containing a pyridinium cation. J Phys Chem B 112: 1473-1476. doi:10.1021/jp076895k. PubMed: 18201076. 18201076

[23] Dyson PJ, Laurenczy G, Ohlin CA, Vallance J, Welton T (2003) Determination of hydrogen concentration in ionic liquids and the effect (or lack of) on rates of hydrogenation. Chem Commun (Camb): 2418-2419. PubMed: 14587710. 14587710 10.1039/b308309h

[24] Fukaya Y, Sugimoto A, Ohno H (2006) Superior solubility of polysaccharides in low viscosity, polar, and halogen-free 1,3-dialkylimidazolium formates. Biomacromolecules 7: 3295-3297. PubMed: 17154453. 17154453 10.1021/bm060327d

[25] Fujita K, Murata K, Masuda M, Nakamura N, Ohno H (2012) Ionic liquids designed for advanced applications in bioelectrochemistry. Rsc Advances 2: 4018-4030. doi:10.1039/c2ra01045c.

[26] Dennison SM, Bowen ME, Brunger AT, Lentz BR (2006) Neuronal SNAREs do not trigger fusion between synthetic membranes but do promote PEG-mediated membrane fusion. Biophys J 90: 1661-1675. doi:10.1529/biophysj.105.069617. PubMed: 16339880. 16339880 PMC1367317

[27] Cromie SR, Del Pópolo MG, Ballone P (2009) Interaction of room temperature ionic liquid solutions with a cholesterol bilayer. J Phys Chem B 113: 11642-11648. doi:10.1021/jp904060y. PubMed: 19655771. 19655771

[28] Tamura K, Nakamura N, Ohno H (2012) Cytochrome c dissolved in 1-allyl-3-methylimidazolium chloride type ionic liquid undergoes a quasi-reversible redox reaction up to 140 degrees C. Biotechnol Bioeng 109: 729-735. doi:10.1002/bit.24357. PubMed: 22038699. 22038699

[29] Zhang QG, Wang NN, Yu ZW (2010) The hydrogen bonding interactions between the ionic liquid 1-ethyl-3-methylimidazolium ethyl sulfate and water. J Phys Chem B 114: 4747-4754. PubMed: 20337406. 20337406 10.1021/jp1009498

[30] Lentz BR, Lee JK (1999) Poly(ethylene glycol) (PEG)-mediated fusion between pure lipid bilayers: a mechanism in common with viral fusion and secretory vesicle release? Mol Membr Biol 16: 279-296. doi:10.1080/096876899294508. PubMed: 10766128. 10766128

[31] Xu W, Cooper IE, Angell CA (2003) Ionic Liquids: Ion Mobilities, Glass Temperatures, and Fragilities. JPhys Chem B 107: 6170-6178.

