# Supplementary figures and images for: The Effect of Hydrophilic Ionic Liquids 1-Ethyl-3-Methylimidazolium Lactate and Choline Lactate on Lipid Vesicle Fusion
Source: PLoS One. 2013 Dec 31;8(12):e85467. doi: 10.1371/journal.pone.0085467 (PMC3877375; doi:10.1371/journal.pone.0085467)

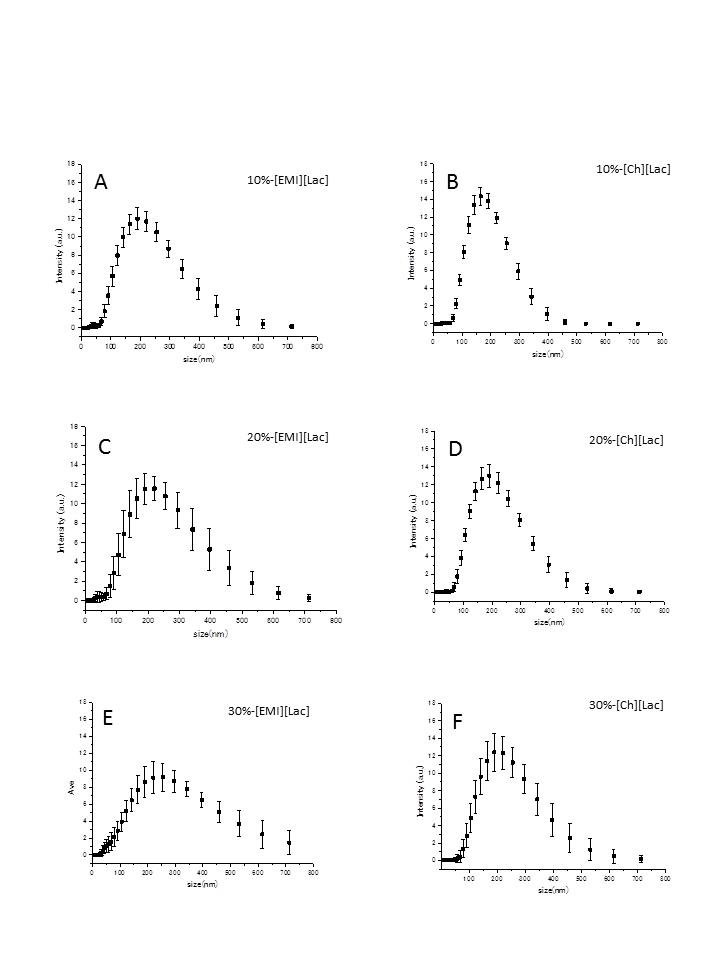

Supplement: Figure S1 — Size distributions of vesicles under various concentrations of ILs. The IL concentrations were 10, 20 and 30% (v/v) in the vesicle solution. For both ILs, the maximum size of the vesicles increased with increasing IL concentration. In addition, the width of the size distribution became broader under increasing IL concentrations. (TIF) [file pone.0085467.s001.tif]
